# Prophylaxis and antibiotic therapy in management protocols of patients treated with oral and intravenous bisphosphonates

**DOI:** 10.4317/jced.53372

**Published:** 2017-01-01

**Authors:** Elena-Beatriz Bermúdez-Bejarano, María-Ángeles Serrera-Figallo, Aida Gutiérrez-Corrales, Manuel-María Romero-Ruiz, Raquel Castillo-de-Oyagüe, José-Luis Gutiérrez-Pérez, Daniel Torres-Lagares

**Affiliations:** 1 Master’s Degree in Oral Surgery. School of Dentistry. University of Seville; 2 Associate Professor. Integrated Dentistry and Patients with Special Diseases. School of Dentistry. University of Seville; 3 Professor of Prostheses. Department of Stomatology. School of Dentistry. Complutense University of Madrid; 4 Professor of Oral Surgery. School of Dentistry. University of Seville

## Abstract

**Introduction:**

Osteonecrosis of the jaw (MRONJ) linked to bisphosphonate treatment has specific characteristics that render its therapeutic management challenging for clinicians. Poor response to standard treatment makes it essential to take special precautions when treating this type of disease; therefore, antibiotic prophylaxis and/or antibiotic therapy have been proposed as effective and helpful tools in these situations.

**Objectives:**

This article seeks to assess published evidence in order to evaluate the different protocols used for antibiotic prophylaxis and/or antibiotic therapy in the general context of patients treated with bisphosphonates.

**Material and Methods:**

A literature review of the last 10 years was carried out in PubMed using the following keywords: “antibiotic prophylaxis and osteonecrosis,” “bisphosphonates AND osteonecrosis AND dental management,” “bisphosphonate AND osteonecrosis AND antibiotic prophylaxis AND oral surgery.” A total of 188 articles were obtained, of which 18 were ultimately selected.

**Results and Discussion:**

In patients treated with oral and intravenous bisphosphonates without chemotherapy-associated osteonecrosis of the jaw, antibiotic prophylaxis prior to oral surgery is an important tool to avoid osteonecrosis and promote healing of the affected area. If the patient previously exhibited chemotherapy-associated osteonecrosis after tooth extraction, antibiotic prophylaxis is indicated to prevent recurrent osteonecrosis and promote healing of the extraction site. If chemotherapy-associated osteonecrosis is already present, antibiotic therapy is a vital part of conservative management to reduce the symptomatology of MRONJ and keep it from worsening. Finally, a lack of clinical data and randomized controlled trials makes it difficult to choose the most appropriate protocol for the various clinical situations studied.

** Key words:**Bisphosphonates, antibiotic prophylaxis, maxillary osteonecrosis, antibiotic treatment.

## Introduction

Antibiotic prophylaxis is used in dentistry to prevent infections in high-risk cases, such as during surgical procedures that enable pathogens to enter the body, or in patients whose general health characteristics or specific medical condition make them more susceptible to contracting new infections (1-5).

Consequently, prophylaxis is used to promote appropriate bioavailability of an antibiotic that can effectively tackle microorganisms and therefore prevent their proliferation and any subsequent infections. This concept is the opposite of antibiotic therapy, which is prescribed in cases of already established infection and is aimed at treating symptoms rather than preventing them (6).

Antibiotic prophylaxis and/or antibiotic therapy protocols have been established as an effective therapeutic tool in the prevention or conservative management of certain diseases. One such disease is chemotherapy-associated osteonecrosis of the jaw caused by bisphosphonates (or other drugs), described by Marx in 2003 (7) as an exposure of necrotic bone with more than eight weeks of evolution, associated with bisphosphonates and no prior radiation therapy. These lesions can progress and become infected or even suffer other complications, making them difficult to manage and with a wide range of therapies providing inconsistent results (8-10).

A distinction should be made between oral and intravenous routes of administration of bisphosphonates (11,12). Intravenous bisphosphonates are indicated for cancer patients (pamidronate and zoledronic acid); these are the most potent and are more likely to result in onset of MRONJ. They increase sevenfold the appearance of MRONJ after performing dental surgery, in comparison with oral bisphosphonates. In addition, it should be noted that the longer a treatment, the higher the risk of developing MRONJ, and larger doses also increase the risk of MRONJ. Intravenous bisphosphonates can remain in the blood for up to 10 years.

The onset of bisphosphonate-related MRONJ has also been correlated with different local factors (extractions, implant placement, periodontal disease, etc.) and systemic factors (endocrine disruptions, tobacco, alcohol, race, age, sex, etc.) (13,14).

In view of these risk factors, patient medical history, and the oral-systemic regions affected by bisphosphonates (including suppressed bone remodeling, deterioration of angiogenesis, toxicity of the soft tissues, modulatory dysfunction of the immune system, and delayed healing), there are many reasons to use antibiotic prophylaxis in patients treated with oral or intravenous bisphosphonates.

On the other hand, patients treated with oral or intravenous bisphosphonates who have already developed chemothera-py-associated osteonecrosis of the jaw may also benefit from antibiotic therapies to avoid potentially serious infection that can worsen symptoms; thus, antibiotic therapy can help improve overall clinical condition (15-17).

The present article seeks to identify the different published protocols of antibiotic prophylaxis and/or antibiotic therapy in patients treated with oral or intravenous bisphosphonates. To this end, a systematic review of the literature was carried out focusing on three clinical situations: a) patients treated with oral or intravenous bisphosphonates and without MRONJ who will undergo a dental extraction, b) patients treated with oral or intravenous bisphosphonates and with previous incidence of MRONJ who will undergo a dental extraction; and c) patients treated with oral or intravenous bisphosphonates and with MRONJ, as part of their conservative management.

## Material and Methods

A review of the literature published in PubMed over the last 10 years was carried out using the following keywords: “antibiotic prophylaxis and osteonecrosis,” “bisphosphonates AND osteonecrosis AND dental management,” “bisphosphonate AND osteonecrosis AND antibiotic prophylaxis AND oral surgery.” The results returned 29, 129, and 13 articles, respectively. The inclusion and exclusion criteria were then applied in order to fulfill the two proposed objectives.

The inclusion criteria for the first proposed objective (antibiotic prophylaxis protocols) were: academic publications written in English that involved reviews of the literature, clinical trials, case control studies, cohorts studies and case series centered on antibiotic prophylaxis (indicating the antibiotic used, doses, time, and dosage used for treatment) in patients treated with oral or intra-venous bisphosphonates, with or without antecedents of developing chemotherapy-associated osteonecrosis of the jaw, who were undergoing oral surgery.

The inclusion criteria for the second proposed objective (antibiotic treatment guidelines) were: academic publications written in English that involved reviews of the literature, clinical trials, or case series that specify the type of antibiotic used, doses and length of treatment, and guidelines used for antibiotic therapy in patients treated with oral and intravenous bisphosphonates with MRONJ, as part of their conservative management (therefore, antibiotics are used as part of patient treatment).

The exclusion criteria for both proposed objectives were: articles that did not meet the eligibility requirements for both proposed objectives, articles unrelated to the research topic, articles that did not have an abstract or with an anonymous author, letters to the editor, and expert opinions.

The selection process was finished by manually searching through all the bibliographic references of the selected articles.

## Results

**Figure 1:**
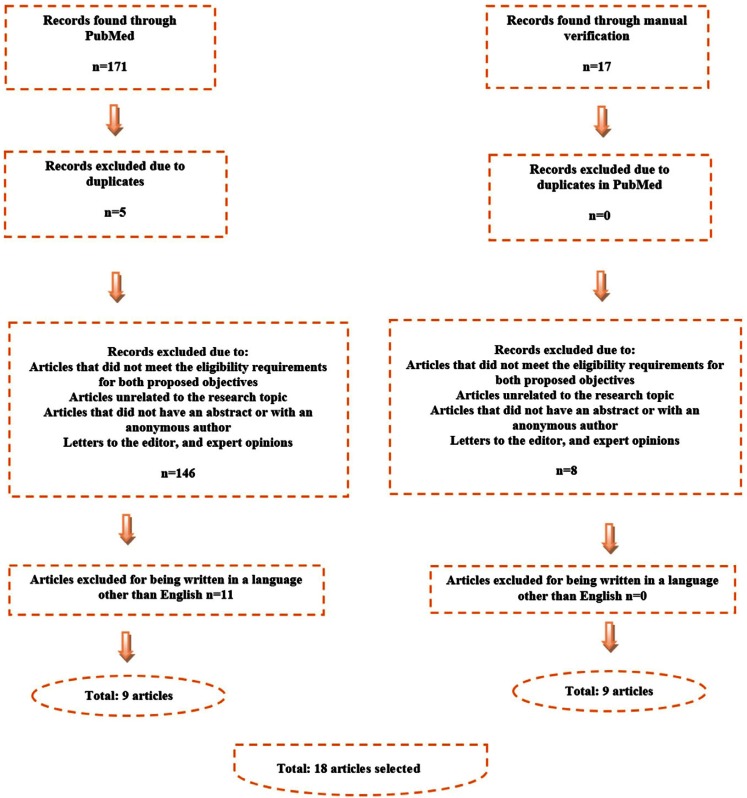
Flow Chart.

The initial search in PubMed yielded 171 results, with an additional 17 articles identified during the manual search of the collected articles’ bibliographic references. The flow chart seen in figure 1 details how the eligibility criteria were applied. A total of 18 articles were selected for final inclusion in the present study.

Results taken from the different articles can be seen in  Table 1 to  Table 3.  Table 1 shows the antibiotic prophylaxis protocols applied to patients without previous MRONJ receiving treatment with oral bisphosphonates. The table also provides information on the antibiotic prophylaxis protocols used in patients without previous MRONJ receiving treatment with intravenous bisphosphonates.  Table 2 is centered on antibiotic prophylaxis protocols in patients treated with oral or intravenous bisphosphonates who had previously had MRONJ. Lastly,  Table 3,  Table 3 continue shows the antibiotic treatment protocols applied within a conservative management approach for patients with MRONJ receiving treatment with both oral and intravenous bisphosphonates.

**Table 1:**
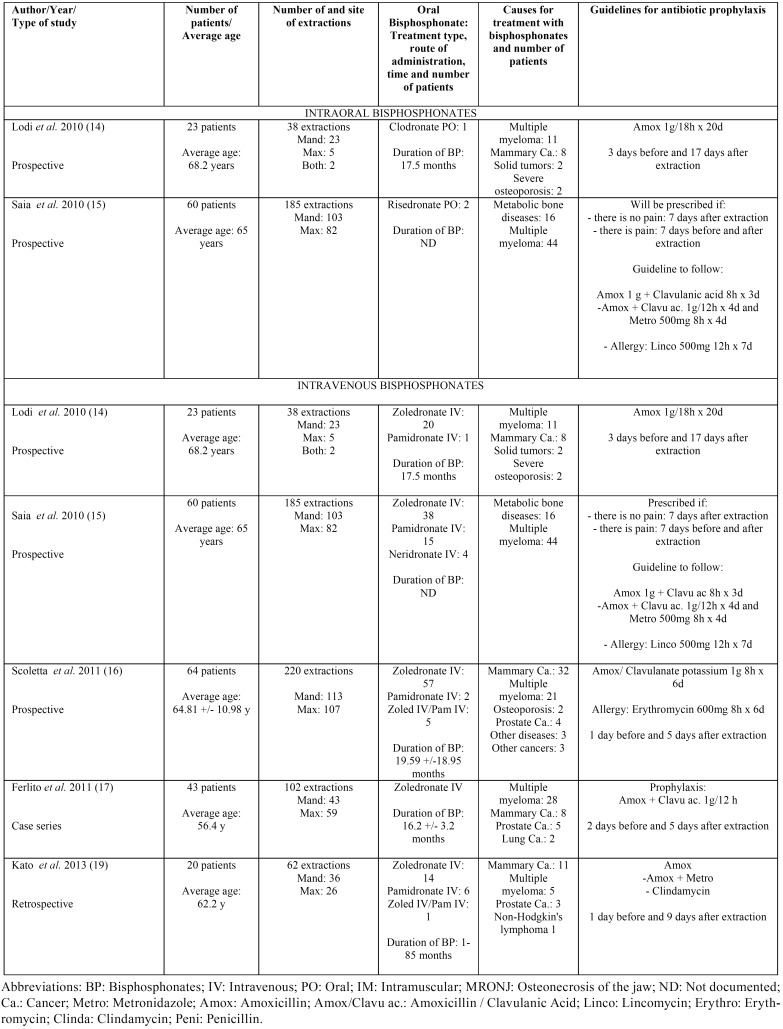
Antibiotic prophylaxis in patients treated with oral and intravenous bisphosphonates without previous MRONJ and who will undergo an extraction.

**Table 2:**
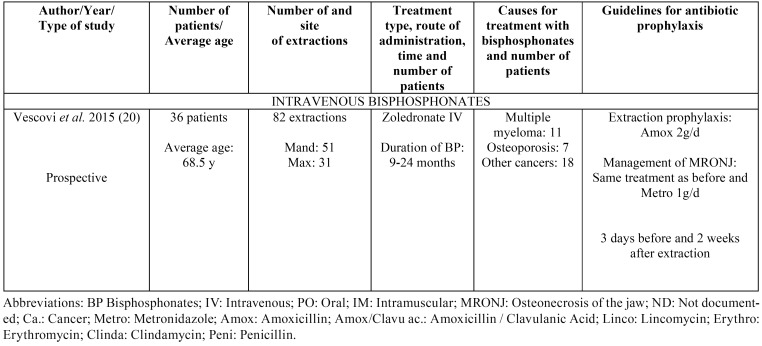
Antibiotic prophylaxis in patients treated with oral (0 articles) or intravenous bisphosphonates without previous MRONJ and who will undergo an extraction.

**Table 3:**
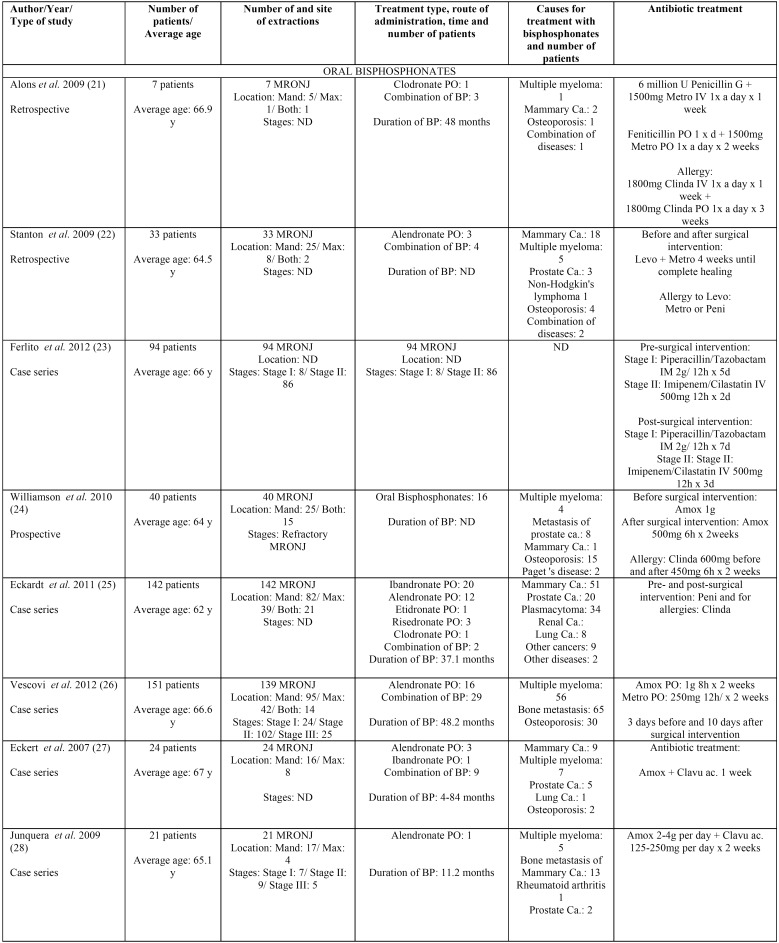
Antibiotic therapy as a conservative management in patients treated with oral and intravenous bisphosphonates with MRONJ.

**Table 3 continue:**
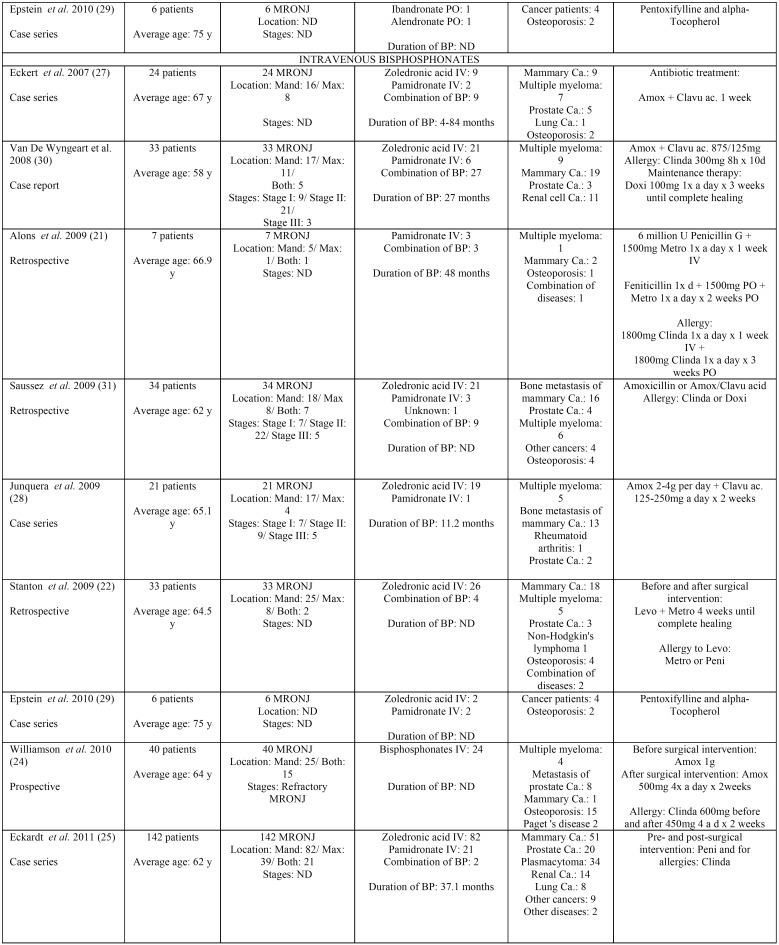
Antibiotic therapy as a conservative management in patients treated with oral and intravenous bisphosphonates with MRONJ.

When antibiotic prophylaxis is used in patients without previous MRONJ who are receiving treatment with oral bisphosphonates ( Table 1), as well as in patients treated with intravenous bisphosphonates ( Table 1), the most frequently used antibiotics are peni-cillin, amoxicillin, amoxicillin/clavulanic acid, metronidazole, and/or a combination thereof. Erythromycin, clindamycin, or even lincomycin are prescribed if the patient is allergic to penicillin or amoxicillin (15).

In the same way, the most widely used antibiotics for the treatment of MRONJ in patients taking oral bisphosphonates are penicillin, amoxicillin, amoxicillin/clavulanic acid, metronidazole, and/or a combination thereof.

With regard to the length of prophylactic antibiotic treatment prior to and following tooth extraction, there is no uniform approach applied in all patients receiving treatment with oral or intravenous bisphosphonates. Despite this, most authors agree that post-extraction treatment regimens in patients receiving oral and intravenous bisphosphonates should be continued until the surgical site has completely healed.

## Discussion

Antibiotic prophylaxis can be beneficial in avoiding the onset of MRONJ in patients who are set to undergo oral surgery (extraction) and are currently being treated with oral and intravenous bisphosphonates. If MRONJ has already developed and is under control, antibiotic treatment prophylaxis can prevent its recurrence. Moreover, antibiotic prophylaxis can help reduce the symptoms of osteonecrosis of the jaw, aiding in a conservative management regimen. However, despite the many studies found in the literature, there is no consensus on which is the most used antibiotic and its dosage (17).

The latest consensus of the American Association of Oral and Maxillofacial Surgeons (AAOMS) (18) refers to the use of antibiotics in the systemic management of such patients in Stage 0 (“Systemic management, including use of pain medication and antibiotics”). Given that the same article identifies this stage as a moment in which there is no clinical evidence of MRONJ, this kind of situation would be the first group of study of our article. Aside from this indication, consensus doesn’t detail the most useful antibiotics or that they should be used. For this reason, clinical experience collected in this review could be useful (18).

Antibiotics used in patients without previous MRONJ who are receiving treatment with oral bisphosphonates, as well as in patients treated with intravenous bisphosphonates ( Table 1), have already been exposed in the Results section. With regard to the length of prophylactic antibiotic treatment prior to and following tooth extraction, there is no uniform approach applied in all patients receiving treatment with oral or intravenous bisphosphonates. Some articles indicate that antibiotics should be prescribed three (14) or seven (15) days before tooth extraction in patients treated with oral bisphosphonates. Similarly, post-extraction recommendations vary, with articles suggesting that antibiotic prophylaxis be administered anywhere from seven (15) to seventeen days (14) post-intervention.

For patients receiving treatment with intravenous bisphosphonates, the articles’ recommendations for when to begin pre-extraction prescription of antibiotics range from one, (16,19) two, (17) or three days before (14) to seven days before extraction (15). Post-extraction antibiotic treatment is recommended to be started five, (16-18) seven, (15) nine, (19) or seventeen days (14) after the procedure. Despite this, most authors agree that post-extraction treatment regimens in patients receiving oral and intravenous bisphosphonates should be continued until the surgical site has completely healed.

For the above reasons, the total length of treatment time can vary greatly. Patients treated with oral bisphosphonates can receive prophylaxis for anywhere from seven (15) to twenty days (14). In patients treated with intravenous bisphosphonates, treatment time can range from six (16) or seven (15,17) to twenty (14) days.

Regarding the use of antibiotics for the treatment of MRONJ, the consensus of the AAOMS (18) identifies as necessary from Stage 2, indicating that the isolated microorganisms are often sensitive to penicillin. Although it indicates a wide variety of antibiotics used to treat MRONJ not identify dose or temporal patterns, which if addressed in this paper, can help fill that consensus. No articles were found on administering antibiotic prophylaxis in patients receiving oral treatment with bisphosphonates who had also suffered previous osteonecrosis of the jaw and were set to undergo an extraction in the near future. Regarding patients undergoing intravenous bisphosphonate treatment, only one article was found, in which the authors prescribe amoxicillin before the extraction and add metronidazole after the procedure (20). The pharmaceutical treatment began three days prior to the procedure and was continued for two weeks afterwards (18 days in total) (20).

Long-term antibiotic treatment appears to yield better results in regard to promoting proper healing of the alveolar sockets (17,20) and avoiding the onset of MRONJ. The most widely used antibiotics for the treatment of MRONJ in patients taking oral bisphosphonates are similar to the used in the first group of clinical situations: penicillin, amoxicillin, amoxicillin/clavulanic acid, metronidazole, and/or a combination thereof. There are also studies that prescribed penicillin G + IV metronidazole, (21) levofloxacin + metronidazole, (22) piperacillin + tazobactam, or imipenem + cilastatin (23). If the patient is allergic to penicillin or amoxicillin, clindamycin is usually prescribed instead (21,24-26,30-32).

There is no consensus on total treatment time. Authors’ recommendations for conservative management of MRONJ using antibiotics include one week, (27) ten days, (26) fifteen days (24,26,28,29), or three or four weeks, until the healing process is complete (21,22). Most of the consulted studies agree that antibiotic treatment should be long-term. This is because depending on the severity of MRONJ, conservative treatment may be accompanied by surgical treatments with varying levels of invasiveness. Consequently, antibiotic therapy is often continued long-term until the clinical remission of signs and symptoms linked to MRONJ or its surgical treatment.

In patients treated with intravenous bisphosphonates, the most commonly used antibiotics are penicillin, amoxicillin, amoxicillin/clavulanic acid, metronidazole, and/or a combination thereof. Some studies also prescribed penicillin G + IV metronidazole, (21) levofloxacin + metronidazole, (22) piperacillin + tazobactam, or imipenem + cilastatin (23). If the patient is allergic to penicillin or amoxicillin, clindamycin is usually prescribed instead (21,24-26,30-32).

No consensus exists on total treatment times, with the approach being similar to treatments described for patients undergoing treatment with oral bisphosphonates.

In any case, it is always best to carry out antibiogram before prescribing any antibiotics; (12) however, broad spectrum antibiotics can be used in cases where MRONJ must be treated as soon as possible.

Sparse clinical data and a lack of randomized controlled trials make it impossible to definitively identify the most appropriate protocol for each of the different clinical situations studied (33,34).

In conclusion, it is clear that in patients being treated with oral and intravenous bisphosphonates who have not had prior chemotherapy-associated osteonecrosis of the jaw, the use of antibiotic prophylaxis prior to oral surgery is an important tool in avoiding osteonecrosis and in promoting proper healing of the affected area. If a patient previously had chemotherapy-associated osteonecrosis after a tooth extraction, antibiotic prophylaxis will be indicated to prevent the recurrence of osteonecrosis and to promote healing of the extraction site. If chemotherapy-associated osteonecrosis is already present, antibiotic therapy is a vital part of conservative management to reduce the symptomatology of MRONJ and keep it from worsening.
